# The Acute Toxicity of Tetrodotoxin and Tetrodotoxin–Saxitoxin Mixtures to Mice by Various Routes of Administration

**DOI:** 10.3390/toxins10110423

**Published:** 2018-10-23

**Authors:** Sarah C. Finch, Michael J. Boundy, D. Tim Harwood

**Affiliations:** 1 AgResearch Limited, Ruakura Research Centre, Private Bag 3123, Hamilton 3240, New Zealand; 2 Cawthron Institute, Private Bag 2, Nelson 7042, New Zealand; michael.boundy@cawthron.org.nz (M.J.B.); tim.harwood@cawthron.org.nz (D.T.H.)

**Keywords:** paralytic shellfish toxins, acute toxicity, oral toxicity, tetrodotoxin, saxitoxin, toxicity equivalence factor

## Abstract

Tetrodotoxin (TTX) is a potent neurotoxin associated with human poisonings through the consumption of pufferfish. More recently, TTX has been identified in bivalve molluscs from diverse geographical environments, including Europe, and is therefore recognised as an emerging threat to food safety. A recent scientific opinion of the EFSA Panel on Contaminants in the Food Chain recognised the need for further data on the acute oral toxicity of TTX and suggested that, since saxitoxin (STX) and TTX had similar modes of action, it was possible that their toxicities were additive so could perhaps be combined to yield one health-based guideline value. The present study determined the toxicity of TTX by various routes of administration. The testing of three different mixtures of STX and TTX and comparing the experimentally determined values to those predicted on the basis of additive toxicity demonstrated that the toxicities of STX and TTX are additive. This illustrates that it is appropriate to treat TTX as a member of the paralytic shellfish group of toxins. Since the toxicity of TTX was found to be the same as STX by feeding, a molar toxicity equivalence factor of 1.0 for TTX can be applied.

## 1. Introduction

Tetrodotoxin (TTX) is a potent neurotoxin, which is well-known for its presence in pufferfish (Fugu), a revered delicacy in Japan [1]. Its name comes from Tetraodontidae, which is the family of pufferfish associated with its presence and currently 22 species of pufferfish of this family are known to contain TTX [2]. Human intoxications and deaths due to TTX in pufferfish are well recognised throughout history, occurring mainly in Japan, China, and Taiwan [3]. Although initially thought to be present solely in pufferfish it was later discovered that TTX was present in a wide range of marine and terrestrial animals including gastropods [4], crabs [5], marine flatworms [6], ribbon worms [7], arrow worms [8], annelid worms [9], starfish [10], grey side-gilled sea slug [11], blue ringed octopus [12], newts [13], frogs [14], and terrestrial flatworms [15]. In addition to intoxication from pufferfish, human poisonings have also been observed due to the ingestion of toxic gastropods and crabs in many countries throughout the world [3]. In more recent times the distribution of pufferfish has become more widespread. This is thought to be due to the opening of the Suez canal and toxic pufferfish have been found in the waters of Turkey, the Israeli coast, Greece and Rhodes Island [16]. Of more concern, TTX has also been identified in bivalve molluscs in diverse geographical environments. In Japan, in 1993, TTX was reported in scallop (*Patinopecten yessoensis*) digestive glands at concentrations of up to 8 mg/kg [17]. In New Zealand, in 2011, TTX was reported in a clam species (pipi; *Paphies australis*) at 0.8 mg/kg [18]. In the UK, in 2014, it was observed in samples of mussels (*Mytilus edulis*) and Pacific oysters (*Crassostrea gigas*) at low concentrations of 0.003–0.12 mg/kg [19]. In Greece, an unexplained positive mouse bioassay screen led to the retrospective analysis of mussels (*Mytilus galloprovincialis*) and clams (*Venus verrucusa*) collected between 2006 and 2012, which were found to contain TTX at concentrations of 0.061–0.194 mg/kg [20]. In China, in 2015, manila clams (*Ruditapes philippinarum*) were found to contain trace levels of TTX [21], and in the Netherlands it was identified in bivalve mollusc samples collected in 2015 and 2016 [22]. This body of evidence clearly demonstrates that TTX is a contaminant of bivalve molluscs and has the potential to pose a food safety risk to consumers. For this reason TTX is recognised as an emerging risk by international regulators.

The question of the origin of TTX remains a controversial one with the two most common hypotheses being that it is being produced by symbiotic bacteria or that it is accumulated through the diet. Although there has been a large amount of work conducted in this area a clear consensus does not yet exist [9,23,24].

**Figure 1:**
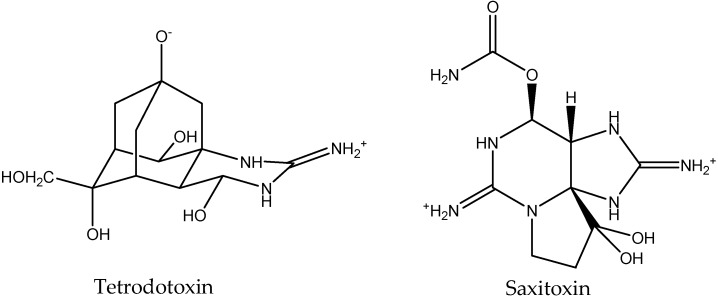
Structures of tetrodotoxin and saxitoxin.

The clinical signs of intoxication by TTX (neuromuscular, gastrointestinal, cardiovascular, and dermatologic symptoms and respiratory distress) are the same as those observed with paralytic shellfish poisoning (PSP), and indeed saxitoxin (STX) and its analogues are found in bivalves [17], pufferfish, crabs, and gastropods along with TTX [25]. Although the chemical structures of TTX and STX are considerably different (Figure 1), both toxins exert their effects through an interaction with voltage-gated sodium channels (Na_v_) [26]. This interaction blocks conductance resulting in inhibition of neuromuscular transmission. Both toxins are active on the α-subunit of the Na_v_ channel although there are some differences in the affinities of TTX and STX for different Na_v_ isoforms [27].

The acute toxicities of STX and its analogues are well-defined [28,29] and a regulatory limit of ≤0.8 mg of STX (2HCl) equivalents/kg of mollusc flesh has been set for this class of marine toxin [30]. This limit has been demonstrated to be appropriate for protecting human health and facilitating international trade. The concentrations of STX and its analogues can be quantified by analytical methods but to translate this into an estimate of toxicity the relative toxicities of the individual components must be applied. These relative toxicities are termed toxicity equivalence factors (TEFs) and are defined as the “toxicity ratio of a compound from a chemical group that shares the same mode of action of a reference compound in the same group.” For the PSP group of toxins, STX is used as the reference compound and is assigned a TEF of 1.0. The acute toxicity of each analogue, on a molar basis, can then be expressed as a fraction of the toxicity of STX to yield TEFs. To determine TEFs, toxicity data is considered with the following order of importance; data from human cases (outbreaks), oral LD_50_ in animals, intraperitoneal (i.p.) LD_50_ in animals, mouse bioassay, and in vitro data [31,32]. Since outbreak data is rarely available, oral toxicity data determined using rodents is usually the best achievable option. This can be determined by dosing purified toxin either by gavage or by feeding. It is well-recognised that feeding is the superior choice as administration by gavage in experiments with rodents gives an overestimation of toxicity. This is because, unlike a human, the stomach contents of rodents is semi-solid such that, when a dose is administered by gavage, it can flow around the semi-solid mass to be rapidly absorbed by the duodenum. In contrast, if the test compound is incorporated with a solid matrix when this is consumed by a rodent, it mixes with the existing stomach contents as occurs with the liquid stomach contents of humans [32,33]. Once the TEFs are available, the overall toxicity of a sample can be calculated by adding the concentration of each compound multiplied by the individual TEF values. There is very little data available on the acute toxicity of TTX and much of what exists lacks adequate experimental detail. Toxicity by i.p. injection was found to be 10.7 [34] and 10 µg/kg [35], which is very similar to that published for STX. The toxicity of TTX by gavage was found to be 532 [34] and 232 µg/kg [36]. A No Observable Adverse Effect Level (NOAEL) of 75 µg/kg was also determined in the latter study.

Some Asian countries have policies to manage the risk of TTX poisoning by pufferfish which is based on a mouse bioassay (MBA). Similarly, a MBA for the PSP toxins has traditionally been used, but neither the PSP MBA nor the TTX MBA can distinguish between STX and TTX since both toxin types induce the same symptomology. The two MBAs follow the same principle, although the TTX MBA uses acetic acid as the extraction and dosing solution [37], whereas the PSP MBA uses a solution of hydrochloric acid [38]. Because TEFs of saxitoxin analogues have previously been determined using 3 mM hydrochloric acid as the solvent vehicle [28,29], this protocol was followed in the current study. However, to ensure that the toxicity of TTX was not influenced by the dosing solvent employed, the LD_50_ of TTX was determined by i.p. injection using both acetic acid as the solvent vehicle, as used in the TTX MBA, and using hydrochloric acid as the solvent vehicle, as used in the PSP MBA and in the toxicological studies of STX analogues. Many countries, including those in Europe and Australasia, have stopped using the MBA for regulatory monitoring and instead use instrumental techniques such as liquid chromatography coupled with fluorescence detection or functional assays such as receptor binding assays. TTX is not currently included on the list of marine biotoxins to be tested either at the EU or international level [30]. The risk posed by toxic pufferfish is managed by the European Union (EU) by banning the sale of fish belonging to the families Tetraodonidae, Molidae, Diodontidae, and Canthigasteridae, including products derived from these fish species [39]. While the STX group of toxins is monitored and regulated within the EU, the presence of TTX will not be routinely detected in shellfish. In New Zealand, LC-MS is used for the quantitative screening of paralytic shellfish toxins, a method that also detects TTX. Due to the recent discovery of TTX-contaminated shellfish in European waters, TTX is considered an emerging threat to food safety. The EFSA Panel on Contaminants in the Food Chain (CONTAM Panel) was asked to deliver a scientific opinion on the risk related to the presence of tetrodotoxin (TTX) and TTX analogues in marine bivalves and gastropods. The panel reviewed all of the available literature and proposed an acute reference dose (ARfD) of 0.25 µg/kg for TTX and its analogues based on the NOAEL determined by Abal et al. [36]. This is lower than the ARfD of STX (0.5 µg/kg) [40]. Using a large portion size of 400 g of bivalves, they then determined that a concentration below 0.044 mg/kg TTX equivalents/kg of shellfish meat was considered not to result in adverse effects in humans [22]. Concentrations in bivalves have been reported that exceed this level, which implies that there is a potential health risk if a large portion size is consumed. The CONTAM Panel recognised the need for further information on the acute oral toxicity of TTX and noted that, “as saxitoxin and tetrodotoxin exert similar toxic effects via a similar mode of action, the possibility to combine saxitoxin and its analogues together with tetrodotoxin and its analogues in one health-based guidance factor (HBGV) should be explored.”

We have addressed these two recommendations by determining the toxicity of TTX by a number of different routes of exposure including feeding and investigating whether the toxicities of STX and TTX are additive. In addition, we have determined NOAELs for TTX and STX by feeding to allow for a better estimation of the ARfD. Results showed that the oral toxicity of TTX by feeding was the same as that of STX and that the toxicities of the two toxin types are additive. This demonstrates that including TTX in the estimation of PSP toxicity is valid. The inclusion of TTX in the estimation of PSP toxicity (as expressed in STX.2HCl equivalents) will ensure that samples of shellfish containing STX and/or TTX above acceptable levels will not be sold to consumers, thereby eliminating the risk to food safety posed by these compounds.

## 2. Results

### 2.1. Purity of TTX and STX

The concentration of the TTX test material was determined using reference materials from NRC and Cifga, which were found to give identical results and showed a high level of purity. The only TTX analogues detected in the TTX test material were 4epi-TTX and 4,9anhydro-TTX. These impurities were quantified using the TTX certified reference material (CRM) (Cifga) (Table 1). The concentration and purity of the TTX material was unchanged over the course of the study.

The STX was shown to be of very high purity with only minor traces of the STX analogues neosaxitoxin (neoSTX) and decarbamoyl saxitoxin (dcSTX) (Table 2).

### 2.2. Determination of the Specific Activities of STX and TTX by the PSP MBA

The specific activity of STX was determined in this study to be 2085 MU/µmol, which is very close to the figure of 2090 MU/µmol, which has previously been reported by Munday et al. [28]. The specific activity of TTX was determined to be 1120 MU/µmol.

### 2.3. Determination of the Correlation between Dose Rate and Death Time for TTX

**Figure 2:**
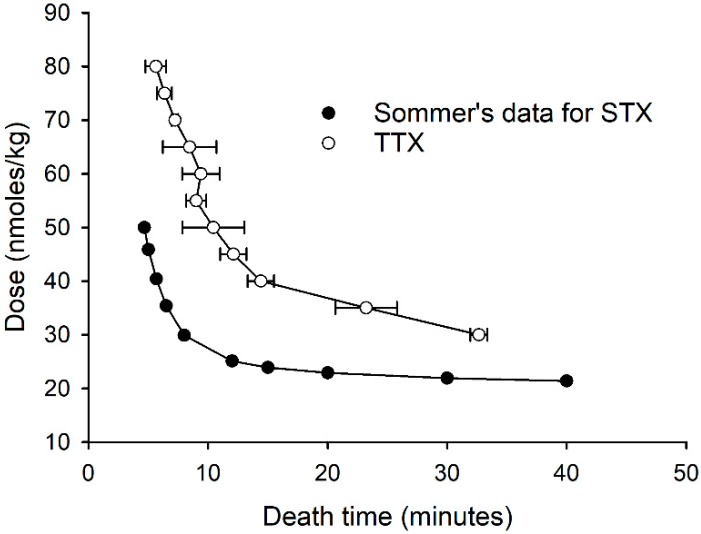
Dose–death time curve of mice injected i.p. with TTX, and the dose–death curve for STX calculated from the data of Sommer.

The relationship between the dose of TTX administered i.p. and the death time (mean ± standard deviation) is presented in Figure 2. Death was observed for all of the mice in the groups dosed 80, 75, 70, 65, and 60 nmol/kg, while 86%, 80%, 83%, 80%, 40%, 40%, and 0% of the mice died in the groups dosed 55, 50, 45, 40, 35, 30, and 25 nmol/kg, respectively (Table S1). For comparison, a dose rate versus death time graph was constructed for STX based on that predicted from Sommer’s table in the AOAC Official Test Method 959.08 [38]. This was calculated from the specific activity determined in this study (2085 MU/µmole) for STX.2HCl (372.21 g/mol). The dose–death time curve derived for TTX showed a similar linearity as that predicted by Sommer’s table at high dose rates of TTX (≥40 nmol/kg). However, at lower dose rates, in contrast to the STX curve, that of TTX did not level out, indicating that the toxicity of TTX would be underestimated if based on the MBA figure.

### 2.4. Determination of Median Lethal Doses by i.p. Injection

The median lethal doses (LD_50_ values) of STX, TTX, and three mixtures containing different ratios of the two toxin types dosed in HCl (3 mM) solutions are presented in Table 3. The clinical signs induced by STX and TTX were indistinguishable. At lethal doses, mice very rapidly became lethargic with abdominal breathing. Within 10 min, the animals became immobile and respiration rates slowed until breathing ceased. Death was observed within 45 min (Table S2). At sub-lethal doses, mice initially showed the same clinical signs of lethargy, immobility, and abdominal breathing. However, mice started to become more alert 30–90 min post-dosing and appeared normal by 4 h. Over the following 14-day observation period, mice exhibited a normal appearance and behaviour, and all treated mice gained weight. No abnormalities were observed at necropsy. If the toxicities of STX and TTX are additive, then the toxicity of a mixture of the two compounds can be predicted using Equation (1).

(1) $${\mathrm{LD}}_{50}\;\mathrm{mixture}=100/\left(\frac{\%\;\mathrm{STX}}{{\mathrm{LD}}_{50}\;\mathrm{STX}}+\frac{\%\;\mathrm{TTX}}{{\mathrm{LD}}_{50}\;\mathrm{TTX}}\right)$$

where LD_50_ mixture = the predicted LD_50_ of the mixture; LD_50_ STX = the experimentally determined LD_50_ of STX; LD_50_ TTX = the experimentally determined LD_50_ of TTX; % STX = the molar contribution of STX to the mixture represented as a percentage of the total; % TTX = the molar contribution of TTX to the mixture represented as a percentage of the total.

The predicted toxicity of the three STX/TTX mixtures was calculated using this equation and then compared to the toxicities that had been experimentally determined (Table 3). These two values showed a good correlation, which demonstrated that the toxicities of STX and TTX were additive when injected i.p.

The LD_50_ of TTX by i.p. injection determined using 0.1% acetic acid as the solvent vehicle was 31.2 nmol/kg with 95% confidence limits of 29.1 and 36.1 nmol/kg. This is in agreement with that determined using 3 mM HCl as the solvent vehicle (31.2 (27.6–35.0) nmol/kg), showing that the toxicity of TTX is not influenced by the type of acidic solution utilised.

### 2.5. Determination of Median Lethal Doses and NOAELs by Oral Administration

The median lethal doses (LD_50_ values) of STX and TTX by gavage and by feeding are presented in Table 4. In addition, the median lethal doses of three mixtures containing different ratios of the two toxin types and the NOAELs of STX and TTX were determined by feeding (Table 4). Oral administration of the mixtures by feeding rather than by gavage was chosen due to the greater accuracy and relevance of this route of exposure. The clinical signs of toxicity induced by STX and TTX when administered orally were indistinguishable. For both toxin types and for the mixtures, mice initially became hunched and lethargic. This symptom was generally present in all of the dosed mice at all of the dose rates tested. Although not always present at the lower dose rates, the movement of more severely affected mice was characterised by the hind legs being splayed. All mice that died and some of the severely affected survivors showed some paralysis as well as whole-body tremors. Respiration rates were slowed as observed with i.p. administration. Death was often associated with jerky, running movements of the back legs. Compared to i.p. administration, the time of onset, death times and recovery times observed with administration by gavage and feeding were extended. Onset of symptoms were observed up to 2½ h post-dosing, death times were observed 1–5 h post-dosing (Tables S3 and S4), and recovery from sub-lethal doses could take up to 7 h. Over the following 14-day observation period, mice exhibited normal appearance and behaviour, and all mice gained weight. No abnormalities were observed at necropsy. The predicted toxicities of the STX/TTX mixtures by feeding were calculated using Equation (1) and were compared to those experimentally determined (Table 4). These values showed a close correlation with the exception of the LD_50_ value determined for the 1:2 STX/TTX mixture, which was higher than that predicted. This outlier result was put down to a greater variation in animal response for this dosing group compared to the others. This hypothesis is consistent with the large 95% confidence limits observed for this particular LD_50_ determination. These results demonstrate that the toxicities of STX and TTX are additive when fed to mice.

For the determination of NOAEL, mice were observed continuously for 3 h, and any change in behaviour, posture, respiration rate, and movement was noted. The NOAELs determined by feeding were very similar for STX and TTX (Table 4). At a dose rate of 1430 nmol/kg, 2/2 mice dosed STX and 2/3 mice dosed TTX showed a change in posture (lying flat rather than in their natural position), were lethargic, and exhibited abdominal breathing. These symptoms occurred 30 min–1 ½ h post-dosing and lasted for 10–45 min. At a dose rate of 1270 nmol/kg, 2/3 mice dosed TTX and 1/3 mice dosed STX showed adverse effects but at a dose rate of 1140 nmol/kgno adverse effects were seen in any mice dosed either STX or TTX. Over the following 14-day observation period, mice exhibited normal appearance and behaviour, and all mice gained weight. No abnormalities were observed at necropsy.

## 3. Discussion

A comparison of the dose–death time curve determined for TTX with that of STX derived from Sommer’s table showed the dose–death time curve shapes to be considerably different. This would result in the toxicity of TTX being underestimated if based on the MBA. The MBA is a bioassay rather than a toxicological evaluation, and its assumption that the relationship between the dose and death time is the same for STX and related compounds is clearly not valid for TTX. This is consistent with the observation that the MBA is not appropriate for estimating the toxicity of other STX analogues [28]. A comparison of the dose–death time curve of TTX with those previously reported in the literature showed it to be very similar to those of decarbamoyl saxitoxin and the equilibrated epimer mixture of gonyautoxins 2 & 3 (70:30 ratio) [28].

As expected, the median lethal dose of TTX was found to be lowest by i.p. injection which is due to rapid and extensive absorption from the peritoneal cavity. Toxicity was found to be greater by gavage in comparison to feeding. This is also a well-recognised phenomenon and has been observed previously for seafood toxins [29]. This is due to the stomach contents of rodents being of a semi-solid consistency, meaning that the gavage dose can bypass this mass to be rapidly absorbed by the duodenum, giving an over-estimation of toxicity. It is interesting to note that the toxicity of TTX relative to STX is dependent on the route of administration (Table 5). This difference is due to the impact of toxicokinetics. By i.p. injection, absorption of the toxin is not required, whereas by oral administration the toxicity is influenced by absorption, by distribution rates throughout the body, and by metabolism. A comparison between the LD_50_ values of other shellfish toxins by i.p. injection and by oral administration shows a wide range of ratios [29]. These results clearly show that the oral toxicity of seafood toxins cannot be predicted on the basis of i.p. toxicity.

The LD_50_ values determined for STX as part of this study (24.0, 1237, and 2850 nmol/kg by i.p., gavage, and feeding, respectively) are consistent with those previously published by Munday et al. (28.8, 1190, and 3200 nmol/kg) [28]. Little reliable toxicity data for TTX is available in the literature. However, although the source and hence the purity of the TTX used was not identified, studies by Xu et al. [34] and Kao and Fuhrman [35] quote LD_50_ values by i.p. injection of 10.7 and 10.0 µg/kg, respectively. When converted to toxicity on a molar basis (33.5 and 31.3 nmol/kg), this shows a close correlation to the result obtained for TTX in this study (31.2 nmol/kg). By gavage, we determined an LD_50_ of 1890 nmol/kg, which is consistent with that of Xu et al. (532 µg/kg; 1667 nmol/kg) but not that of Abal et al. (232 µg/kg; 727 nmol/kg) [36]. There are a number of possible explanations for this difference. Firstly, the protocol employed by Abal et al. used mice, which had been fasted for 12 h, much longer than the 3–4 h specified in the OECD guideline [41]. Whether to fast animals or not prior to testing is long debated, but we chose to use fed mice, as this better represents reality, as seafood is unlikely to be consumed on an empty stomach. Furthermore, the dosing of animals early in the morning, which is possible without fasting, allows close observation for 10 h enabling an ethical decision to be made over whether an animal is likely to endure prolonged distress. Another significant difference between the two protocols is the duration of the experiments. In the Abal et al. study, the experiment was only 2 h in duration and any animals surviving this period were killed, which negated the possibility of deaths at later time points, a concern noted by the EFSA CONTAM Panel [22]. In our study, we observed mice for the full 14-day period as specified in the OECD guideline, and we observed deaths in mice up to 7 h post-dosing. This observation is consistent with the prolonged deaths observed by Vlamis et al. [20], with extracts of TTX-containing shellfish. The death times quoted in the Abal et al. study were surprisingly short, occurring in a matter of minutes in a number of instances. In contrast, we did not see onset of symptoms before 15 min post-dosing in any of our mice dosed by gavage or by feeding, even at high dose rates. Many researchers have highlighted the difficulty of dosing by gavage and how different operators can influence the result [42]. The LD_50_ and NOAEL determined for TTX in this study by feeding rather than by gavage therefore represent the most accurate data available for use in the assessment of risk posed by TTX-contaminated seafood.

The ARfD determined for TTX by the EFSA CONTAM Panel was calculated on the basis of the NOAEL of 75 µg/kg (235 nmol/kg) published by Abal et al. [36], which was generated by gavage. As already discussed, gavage administration to mice is fraught with uncertainty, and we consider the NOAEL of 1294 nmol/kg determined in this study by feeding to be more robust. If we apply the same logic as that used by the EFSA CONTAM Panel, we can calculate an updated ARfD. This is achieved by using the dose rate one step lower in the dose progression sequence (1010 nmol/kg) used in our study as the reference point and applying an uncertainty factor of 100. This results in an ARfD for TTX of 10.1 nmol/kg (3.2 µg/kg). This figure is almost 13 times higher than that proposed by the EFSA CONTAM Panel [22]. Again, following the logic of the panel, based on a large portion size (400 g) and an adult body weight of 70 kg, the 3.2 µg/kg ARfD yields a figure of 560 µg TTX/kg of shellfish meat, which would not be expected to lead to adverse effects in humans. For STX, an ARfD of 0.5 µg/kg (1.34 nmol/kg), which is based on human poisoning data, has been proposed [40]. In our study we found that the NOAELs of STX and TTX are the same (1279 and 1294 nmol/kg, respectively) so alternatively the ARfD of TTX could be set at the same level as that of STX (1.34 nmol/kg), which equates to 0.43 µg/kg for TTX.

However, rather than treating STX and TTX separately, it would be much simpler and accurate to combine them together since it is the total risk posed by shellfish products, which is of importance. The results presented demonstrate that the toxicities of TTX and STX are additive. This means that STX and its analogues and TTX and its analogues can be combined to yield one HBGV. To determine the overall toxicity of seafood samples containing STX and/or TTX, the concentrations of each individual analogue can be measured by analytical methods, such as those developed using LC-MS [43,44]. To yield toxicity information, the relative toxicity of each compound must then be applied, which requires the determination of TEFs. The best data for determining TEFs is oral toxicity by feeding. The LD_50_ determined for TTX by feeding in the present study is therefore the most relevant data for establishing the TEF and gives a value of 1.0 on a molar basis for TTX.

The threat of TTX-contaminated seafood to consumers is becoming of increased importance due to the observation of TTX in a greater number of seafood types over wider geographical areas. The incorporation of TTX into the toxicity assessment of shellfish for the PSP group of toxins will mean that it will be built into the maximum level of ≤0.8 mg of STX (2HCl) equivalents/kg of mollusc flesh, which will ensure that shellfish is safe for human consumption. Further work is required to determine TEFs by feeding for TTX analogues observed in bivalve species.

## 4. Materials and Methods

### 4.1. Purity and Quantity Assessment of TTX and STX

TTX citrate free (10 mg; Cayman Chemicals, purchased from Sapphire Bioscience, Redfern, NSW, Australia) was dissolved in 10 mL of 10 mM acetic acid. This solution was calibrated against certified reference material from the National Research Council of Canada (NRC) and from Cifga (Lugo, Spain) using high performance liquid chromatography with ultra-violet detection (HPLC-UV) and liquid chromatography with tandem quadrupole mass spectrometry (LC-MS/MS). HPLC-UV was performed using an Agilent Zorbax Bonus-RP 3.5 µm, 4.6 × 150 mm column (Agilent, Santa Clara, CA, USA) at 20 °C, which was eluted with a mobile phase of 11 mmol heptanesulfonate with 2.7 mmol phosphoric acid (pH 7.1) and a flow rate of 1 mL/min. Eluting compounds were detected at 210 nm using a photodiode array detector. The impurities were determined and quantified by LC-MS/MS. LC-MS/MS was performed using a Waters Xevo TQ-S (Waters, Milford, MA, USA) with Waters Acquity i-Class UPLC (Waters, Milford, MA, USA) in accord with the method of Boundy et al. [43]. MRM transitions for TTX analogues monitored in positive electrospray ionisation mode used are shown in Table 6.

STX was supplied by Cawthron Natural Compounds (CNC, NZ), and calibrated against paralytic shellfish toxin certified reference materials from NRC. Purity of the STX test material was determined using HPLC-UV. Liquid chromatography was performed using an Agilent Zorbax Bonus-RP 3.5 µm, 4.6 × 150 mm column (Agilent, Santa Clara, CA, USA) at 20 °C, which was eluted with a mobile phase of 11.5% acetonitrile with 11 mmol heptanesulfonate and 16.5 mmol phosphoric acid (pH 7.1) and a flow rate of 1 mL/min. Eluting compounds were detected at 210 nm using a photodiode array detector. Concentration and purity were determined by LC-MS/MS monitoring of MRM transitions on a Shimadzu LCMS-8050 (Shimadzu, Kyoto, Japan) coupled with Shimadzu Nexera X2 (Shimadzu, Kyoto, Japan) (Table 7). Positive and negative electrospray ionisation modes were used (ESI+, ESI−). Ionisation voltage: 0.5 kV, −0.5 kV. Nebulisation gas flow 2 L/min, heating gas: 10 L/min, drying gas: 10 L/min, interface temperature: 300 °C, desolvation line temperature: 250 °C, heat block temperature: 400 °C, Mobile Phase A: 0.1% acetic acid in water, Mobile Phase B: 0.1% acetic acid in acetonitrile. Flow rate was 0.6 mL/min with a Waters Acquity BEH Amide 1.7 µm 2.1 × 100 mm column (Milford, MA, USA) at 60 °C. Initial conditions were 80% B, held for 6 min, followed by a linear gradient from 80% B to 55% B over 5 min, then returned to 80% B over 0.5 min, and held and re-equilibrated for 3.5 min.

Working solutions of the toxins were prepared gravimetrically by taking weighed aliquots of the stock solutions and diluting with 3 mM HCl in accord with previous studies [29].

### 4.2. Animals

Female Swiss albino mice (18–22 g) were bred at AgResearch, Ruakura, New Zealand. The mice were housed individually during the experiments and were allowed unrestricted access to food (Rat and Mouse Cubes, Speciality Feeds Ltd., Glen Forrest, Western Australia) and water. All experiments were approved by the Ruakura Animal Ethics Committee established under the Animal Protection (code of ethical conduct) Regulations Act, 1987 (New Zealand), Project Number 14320, approval date 2 November 2017.

### 4.3. Determination of the Specific Activities of STX and TTX by the PSP MBA

The specific activities of TTX and STX were determined by taking aliquots of the standard solutions and diluting to 1 mL with 3 mM HCl. These solutions were then injected i.p. into mice according to the protocol of AOAC Official Test Method 959.08 [38]. In brief, dose rates were used to yield death times of 5–7 min and the median death times calculated. Using Sommer’s table in the AOAC method, the death time could then be converted to mouse units and the specific activities (MU/µmol) calculated.

### 4.4. Determination of the Correlation between Dose Rate and Death Time for TTX

A dose versus death time graph was constructed by i.p. injecting groups of mice (5–7 animals) with TTX solutions (aliquots of standard solutions diluted to 1 mL with 3 mM HCl) at dose rates of between 25 nmol/kg and 80 nmol/kg with 5 nmol/kg increments. These dose rates spanned a concentration range that induced death within 6 min and that which resulted in no deaths in any mice of the dosing group.

### 4.5. Determination of Median Lethal Doses

Acute toxicities were determined according to the principles of OECD guideline 425 [41]. This guideline keeps the number of animals used to a minimum while still yielding a robust determination of the median lethal dose and an estimate of confidence intervals. It employs an up-and-down procedure whereby one animal is given a dose of the test compound at one step below the estimated LD_50_. If this animal survives, the dose for the next animal is increased by a factor determined by the computer program associated with the guideline [45]. This factor is determined from an estimate of the slope steepness of the dose–response curve. If the initial animal dies, the dose for the next animal is decreased by the same factor. Dosing is continued until 4 live–death reversals have been achieved.

Mice were weighed immediately prior to dosing and the appropriate quantities of test compounds calculated to yield the required doses on a µmol/kg basis. Aliquots of the test compounds were diluted with 3 mM HCl. A dosing volume of 1 mL was used for i.p. injection and a dosing volume of 200 µL was used for administration by gavage. The acute toxicity of TTX by i.p. injection was also determined using 0.1% acetic acid as the solvent vehicle and a dosing volume of 1 mL. For oral dosing, mice were trained to eat small quantities of cream cheese [33]. This was done by first feeding groups of weanling mice twice a day with cream cheese (~1 g). When mice were happy to eat this matrix, they were split into individual cages where the twice-daily feeding continued (~200 mg). At the time of dosing, aliquots of the test compound were mixed with cream cheese (150 mg) on a watch glass. Mice consumed the laced cream cheese within 30 s. To avoid any diurnal variations in response, all dosing was conducted between 8:00 and 9:30 a.m. All mice were monitored intensively during the day of dosing. Mice that survived were monitored for a 14-day period which included a measurement of body weight. After 14 days, the animals were killed by carbon dioxide inhalation and necropsied.

### 4.6. Determination of NOAEL

Mice were dosed by feeding according to the methods described above. However, rather than using death as the parameter, “toxic effect” was instead used. Toxic effect was determined by observing mice continuously for 3 h and noting any change in posture, respiratory rate, or movement.

## Figures and Tables

**Table 1:** Relative molar contribution of detected analogues in the tetrodotoxin (TTX) test material.

Compound Name	% Contribution
TTX	99.54%
4epi-TTX	0.19% ^1^
4,9-anhydro-TTX	0.27%

^1^ 4epi-TTX concentration was determined on the basis of the Cifga TTX CRM although this analyte is not certified.

**Table 2:** Relative molar contribution of detected analogues in the saxitoxin (STX) test material.

Compound Name	% Contribution
STX	99.79%
neoSTX	0.05%
dcSTX	0.16%

**Table 3:** Predicted and experimentally determined acute toxicities of test compounds by i.p. injection.

Compound	Predicted LD_50_	LD_50_ (nmol/kg) ^1^
STX		24.0 (22.1–24.8)
TTX		31.2 (27.6–35.0)
STX/TTX (1:2)	28.4	27.8 (23.3–32.0)
STX/TTX (1:1)	27.1	24.0 (22.1–24.8)
STX/TTX (2:1)	26.0	24.8 (21.6–29.6)

^1^ Figures in brackets indicate 95% confidence limits.

**Table 4:** Predicted and experimentally determined acute toxicities and NOAELs of the test compounds by gavage and by feeding.

Compound	LD_50_ by Gavage (nmol/kg) ^1^	Predicted LD_50_ by Feeding	LD_50_ by Feeding (nmol/kg) ^1^	NOAEL by Feeding (nmol/kg) ^1^
STX	1237 (1056–1630)		2850 (2468–3390)	1270 (1189–1470)
TTX	1890 (1669–2120)		2850 (2475–3410)	1294 (888–1480)
STX/TTX (1:2)	ND	2850	3532 (3016–7830)	ND
STX/TTX (1:1)	ND	2850	2850 (2382–3280)	ND
STX/TTX (2:1)	ND	2850	2850 (2475–3410)	ND

^1^ Figures in brackets indicate 95% confidence limits; ND, Not determined.

**Table 5:** Relative molar LD_50_ values of STX and TTX by different routes of exposure.

Compound	Relative LD_50_ by i.p.	Relative LD_50_ by Gavage	Relative LD_50_ by Feeding
Saxitoxin	1.0	1.0	1.0
Tetrodotoxin	0.77	0.65	1.0

**Table 6:** MRM transitions used for monitoring TTX analogue impurities.

Analogue(s)	Precursor Mass (*m*/*z*)	Product Mass (*m*/*z*)	Collision Energy
11-oxo-TTX (hydrated aldehyde)	336.1	318.1	25
	336.1	162.1	35
(4/6-epi)-TTX	320.1	302.1	26
	320.1	162.1	38
11-oxo-TTX (aldehyde)	318.1	300.1	20
	318.1	162.0	30
11-norTTX-6,6-diol	306.1	288.1	25
	306.1	60.0	35
(5/11)-deoxy-TTX	304.1	286.1	35
	304.1	240.1	35
	304.1	176.1	35
4,9-anhydro-TTX	302.1	256.1	35
	302.1	162.1	35
(4-epi)-11-nor TTX-6S-ol (4-epi)-11-nor TTX-6R-ol	290.1	272.1	26
	290.1	226.0	30
	290.1	60.1	35
5,11-dideoxyTTX 6,11 dideoxyTTX 4,9-anhydro-11-norTTX-6,6-diol	288.1	270.1	25
	288.1	162.1	35
	288.1	60.1	35
4,9-anhydro-5-deoxy-TTX iso-anhydro-deoxy-TTX 4,9-anhydro-11-deoxy-TTX	286.1	135.1	30
	286.1	60.1	25
(4-epi)-5,6,11-trideoxyTTX 4,9-anhydro-11-norTTX-6(S/R)-ol	272.1	254.1	20
	272.1	95.0	35
	272.1	60.0	35
4,9-anhydro-5,11-dideoxyTTX 4,9-anhydro-6,11-dideoxyTTX Iso-anhydro-dideoxy-TTX	270.1	176.1	25
	270.1	166.1	25
	270.1	60.1	25
4,9-anhydro-5,6,11-trideoxy-TTX	254.1	208.1	26
	254.1	60.1	35

**Table 7:** MRM transitions used for monitoring saxitoxin analogue impurities.

Analogue(s)	Ionisation Mode	Precursor (*m*/*z*)	Product (*m*/*z*)	Q1 Prebias (V)	Collision Energy	Q3 Prebias (V)
C3,4	ESI−	490.1	122.0	11	33	12
C1,2	ESI−	474.1	122.0	11	29	21
GTX1,4	ESI−	410.1	367.1	12	15	27
GTX2,3	ESI−	394.1	351.1	12	16	27
GTX6	ESI−	394.1	122.0	19	22	13
GTX5	ESI−	378.1	122.0	14	23	24
dcGTX1,4	ESI−	367.1	349.1	13	18	15
dcGTX2,3	ESI−	351.1	333.1	13	18	15
NEO	ESI+	316.1	126.0	−22	−24	−24
STX	ESI+	300.1	204.1	−15	−24	−14
dcNEO	ESI+	273.1	225.1	−13	−22	−15
dcSTX	ESI+	257.1	126.0	−13	−21	−23

## Supplementary Materials

The following are available online at http://www.mdpi.com/2072-6651/10/11/423/s1. Table S1: Dose and death times for mice dosed tetrodotoxin by intraperitoneal injection; Table S2: Mortalities and death times of mice dosed with tetrodotoxin (TTX), saxitoxin (STX), or mixtures of the two toxins by intraperitoneal injection. Table S3: Mortalities and death times of mice dosed with tetrodotoxin (TTX) or saxitoxin (STX) by gavage. Table S4: Mortalities and death times of mice dosed with tetrodotoxin (TTX), saxitoxin (STX), or mixtures of the two toxins by feeding.
