# Supplementary material for: The Acute Toxicity of Tetrodotoxin and Tetrodotoxin–Saxitoxin Mixtures to Mice by Various Routes of Administration
Source: Toxins (Basel). 2018 Oct 23;10(11):423. doi: 10.3390/toxins10110423 (PMC6266834; doi:10.3390/toxins10110423)
Supplement: Supplementary file 1 [file toxins-10-00423-s001.pdf]

Supplementary Materials: The Acute Toxicity of Tetrodotoxin and Tetrodotoxin–Saxitoxin Mixtures to Mice by Various Routes of Administration

Sarah C. Finch, Michael J. Boundy and D. Tim Harwood

Table S1. Dose and death times for mice dosed tetrodotoxin by intraperitoneal injection.

Dose (nmol/kg)	Total Number of Mice	Number of Deaths	Death Times (min)
80	5	5	5.25, 5.95, 5.33, 4.67, 6.95
75	5	5	5.66, 7.33, 6.25, 6.50, 6.06
70	5	5	6.82, 7.40, 7.33, 7.50, 7.10
65	6	6	12.66, 7.21, 8.92, 7.66, 6.25, 8.00
60	6	6	11.67, 8.50, 9.92, 10.50, 7.55, 8.20
55	7	6	10.25, 9.83, 8.25, 8.42, 8.88, 8.42
50	5	4	8.33, 9.33, 9.88, 14.20
45	6	5	10.33, 12.83, 12.58, 13.05, 11.80
40	5	4	13.95, 15.48, 13.08, 15.13
35	5	2	25.03, 21.40
30	5	2	33.15, 32.15
25	5	0	-

Table S2. Mortalities and death times of mice dosed with tetrodotoxin (TTX), saxitoxin (STX) or mixtures of the two toxins by intraperitoneal injection.

Dose (nmol/kg)	Total Number of Mice	Number of Deaths	Death Times (min)
**STX**			
22.1	3	0	-
24.8	2	2	11.43, 24.45
**TTX**			
24.8	1	0	-
27.8	2	0	-
31.2	3	2	22.10, 45.00
35.0	1	1	19.98
**STX/TTX (1:2)**			
24.8	2	0	-
27.8	3	2	21.25, 33.0
31.2	1	1	20.0
**STX/TTX (1:1)**			
22.1	3	0	-
24.8	2	2	43.67, 30.21
**STX/TTX (2:1)**			
22.1	1	0	-
24.8	3	1	30.0
27.8	2	2	25.9, 15.0

Table S3. Mortalities and death times of mice dosed with tetrodotoxin (TTX) or saxitoxin (STX) by gavage.

Dose (nmol/kg)	Total Number of Mice	Number of Deaths	Death times (min)
**STX**			
1060	3	0	-
1190	4	2	194, 300
1340	3	2	60, 240
1500	1	1	95
**TTX**			
1500	1	0	-
1680	2	0	-
1890	3	2	120, 60
2120	1	1	90

Table S4. Mortalities and death times of mice dosed with tetrodotoxin (TTX), saxitoxin (STX) or mixtures of the two toxins by feeding.

Dose (nmol/kg)	Total Number of Mice	Number of Deaths	Death Times (min)
**STX**			
2540	1	0	-
2850	5	2	90, 360
3200	2	2	98, 190
**TTX**			
2540	1	0	-
2850	3	1	90
3200	2	2	65, 100
**STX/TTX (1:2)**			
2850	2	0	-
3200	3	1	74
3590	3	1	109
4030	2	2	90, 120
**STX/TTX (1:1)**			
2540	2	0	-
2850	3	2	90, 225
3200	1	1	230
**STX/TTX (2:1)**			
2540	1	0	-
2850	3	1	208
3200	2	2	105, 230
